# Exploring the Potential of Artificial Intelligence for Hydrogel Development—A Short Review

**DOI:** 10.3390/gels9110845

**Published:** 2023-10-25

**Authors:** Irina Negut, Bogdan Bita

**Affiliations:** 1 National Institute for Laser, Plasma and Radiation Physics, 409 Atomistilor Street, 077125 Magurele, Romania; negut.irina@inflpr.ro; 2 Faculty of Physics, University of Bucharest, 077125 Magurele, Romania

**Keywords:** hydrogel design, artificial intelligence, machine learning

## Abstract

AI and ML have emerged as transformative tools in various scientific domains, including hydrogel design. This work explores the integration of AI and ML techniques in the realm of hydrogel development, highlighting their significance in enhancing the design, characterisation, and optimisation of hydrogels for diverse applications. We introduced the concept of AI train hydrogel design, underscoring its potential to decode intricate relationships between hydrogel compositions, structures, and properties from complex data sets. In this work, we outlined classical physical and chemical techniques in hydrogel design, setting the stage for AI/ML advancements. These methods provide a foundational understanding for the subsequent AI-driven innovations. Numerical and analytical methods empowered by AI/ML were also included. These computational tools enable predictive simulations of hydrogel behaviour under varying conditions, aiding in property customisation. We also emphasised AI’s impact, elucidating its role in rapid material discovery, precise property predictions, and optimal design. ML techniques like neural networks and support vector machines that expedite pattern recognition and predictive modelling using vast datasets, advancing hydrogel formulation discovery are also presented. AI and ML’s have a transformative influence on hydrogel design. AI and ML have revolutionised hydrogel design by expediting material discovery, optimising properties, reducing costs, and enabling precise customisation. These technologies have the potential to address pressing healthcare and biomedical challenges, offering innovative solutions for drug delivery, tissue engineering, wound healing, and more. By harmonising computational insights with classical techniques, researchers can unlock unprecedented hydrogel potentials, tailoring solutions for diverse applications.

## 1. Introduction: The Concept of AI in Hydrogel Design

Hydrogels are three-dimensional, crosslinked polymer networks that can absorb and retain large amounts of water or biological fluids. They have gained considerable attention in the medical field due to their unique properties, such as high-water content, biocompatibility, and the ability to be tailored to specific applications. The term “hydrogel” is derived from the combination of “hydro”, meaning water, and “gel”, indicating a semi-solid, jelly-like state. This defining characteristic of hydrogels allows them to mimic the soft and hydrated environment of living tissues, making them highly compatible with biological systems. They can be fabricated from polymer chains linked by physical interactions or chemical bonds, allowing for precise control of the degradation rate, porosity, and release profile [1]. Moreover, hydrogels can undergo self-assembly using self-complementary amphiphilic peptides, enabling customisation to achieve optimal geometry for implantation or injection. These appealing features position hydrogels as attractive therapeutic delivery materials, with the potential to encapsulate agents within their water-swollen network. Hydrogels are extensively employed as drug delivery vehicles due to their capacity to encapsulate and release therapeutic agents in a controlled and sustained manner. This controlled drug release enhances treatment efficacy, reduces side effects, and improves patient compliance, particularly in chronic conditions [2]. Additionally, certain hydrogel types possess inherent antibacterial properties [3]. In tissue engineering, hydrogels serve as scaffolds for growing cells and regenerate damaged or lost tissues [4]. Their high-water content and biocompatibility mimic the natural extracellular environment, facilitating cell growth, proliferation, and differentiation [5]. This is particularly significant in the development of artificial organs and in repairing damaged tissues [4]. Moreover, hydrogels play a vital role in wound care and healing. They can maintain a moist environment, which accelerates the wound-healing process, reduces the risk of infection, and minimises scarring [6].

Hydrogels are the material of choice for contact lenses due to their water-retaining properties, ensuring comfort and optical clarity for the wearer [7].

Other applications of hydrogels include diagnostic assays and biosensors [8]. Their ability to undergo volume changes in response to specific analytes, such as glucose or pH, makes them valuable components in various diagnostic applications [8].

While the potential applications of hydrogels in the biomedical and pharmaceutical fields are vast, designing hydrogels with precise properties tailored to each application is a complex endeavour. Researchers must consider factors such as biocompatibility, mechanical strength, degradation rates, and drug release profiles. The interplay of these variables makes hydrogel design challenging and often reliant on time-consuming trial-and-error approaches. Big data generated from experiments, simulations, and computational calculations has provided potential for applying data-driven methodologies in material science, which shows promise for expediting the discovery and design of new materials. This approach harnesses the power of vast datasets generated through experiments, simulations, or observations to gain insights, make predictions, and guide decision-making in the field of materials science and in the context of hydrogel design.

In the 1990s and early 2000s, the integration of AI in hydrogel research began with the application of computational simulations and modelling techniques. Researchers started to use computational methods to simulate hydrogel behaviour under various conditions, aiding in predicting swelling properties, mechanical responses, and drug release kinetics. Hydrogel theoretical modelling is based on continuum mechanical concepts such as balancing laws, kinematics, and constitutive equations. The Flory–Rehner theory, which explains the swelling equilibrium of gels [9], represents a suitable example. As a result of the advancement of computing capabilities, the computational science paradigm gained enormous popularity. Simulations on both the macro- and micro-scales, such as those using the finite element and volume methods, are now possible [10].

These early efforts laid the groundwork for using AI to unravel the complex interactions within hydrogel networks.

As Machine Learning (ML) algorithms advanced, the hydrogel research community recognised the potential of AI to revolutionise material discovery. The utilisation of ML techniques gained traction. These approaches enabled researchers to analyse large datasets, correlate structure–property relationships, and accurately predict hydrogel behaviour. AI algorithms can analyse patient-specific data, such as genetic information, metabolism rates, and medical histories [11], to design hydrogels that deliver drugs with precision. For instance, in cancer treatment, AI-driven hydrogel formulations can adapt drug release rates based on the tumour’s response to therapy, minimising side effects and maximising effectiveness [12]. AI models can simulate and predict the behaviour of hydrogels under various conditions, saving researchers significant time and resources. For example, they can predict how a hydrogel will swell, degrade, or release drugs in response to changes in pH, temperature, or biological factors [13]. AI-driven material design can identify the ideal combination of polymers, crosslinkers, and additives to create hydrogels with specific mechanical, thermal, and chemical properties. This accelerates the development of hydrogels tailored for applications such as wound healing, contact lenses, or tissue scaffolds. High-throughput screening, guided by AI algorithms, enables the evaluation of vast libraries of hydrogel formulations [14]. This accelerates the discovery of hydrogels with desirable properties, reducing the time needed to bring innovative materials to market.

Therefore, this era marked a significant shift from traditional empirical methods to data-driven approaches, offering faster and more informed decision-making in hydrogel design.

Artificial Intelligence (AI) represents a field that involves the development of algorithms and models that enable computers to mimic human intelligence. In hydrogel development, AI offers a novel approach to tackle the challenges associated with hydrogel properties and performance by leveraging data-driven insights, predictive modelling, and optimisation techniques. These challenges are multifaceted and include tailoring hydrogels for precise applications, optimising their mechanical and chemical characteristics, and navigating the complex interplay of material variables. First and foremost, AI expedites the process of discovering new hydrogel formulations. Traditional methods often involve extensive trial-and-error experimentation, consuming considerable time and resources. In contrast, AI-driven algorithms can rapidly analyse vast datasets, predict material properties, and recommend optimal compositions. This acceleration of the research process is particularly crucial in the field of hydrogels, where materials must meet precise criteria for applications in medicine and biology.

AI also offers a level of precision and customisation that was previously unattainable. Researchers can input specific characteristics they desire in a hydrogel—such as mechanical strength, porosity, or biodegradability—into ML models. AI then provides tailored recommendations for material compositions and processing techniques to achieve these desired properties. This level of precision is invaluable when designing hydrogels for diverse applications, from drug delivery systems to tissue engineering scaffolds.

Hydrogel research is inherently complex, involving intricate relationships among various factors. AI can help in this aspect by handling multidimensional data effectively. It identifies patterns and correlations that may elude traditional analysis, allowing researchers to make informed decisions about material design.

In addition, AI integration offers cost and resource efficiencies. It reduces the need for extensive laboratory experimentation, thus saving costs related to materials, equipment, and personnel. Moreover, it minimises material wastage, aligning research practices with environmental sustainability goals.

Interdisciplinary collaboration is another hallmark of AI-driven hydrogel research. This technology bridges the expertise of materials scientists, chemists, biologists, and computer scientists. Their collective knowledge and insights foster innovative solutions to complex challenges in hydrogel development.

AI also enables data-driven insights that can lead to breakthroughs and innovations. By analysing extensive datasets, it uncovers hidden patterns and relationships, guiding researchers toward novel solutions that might otherwise remain undiscovered.

In the pursuit of personalised medicine, AI plays a pivotal role by recommending hydrogel formulations tailored to individual medical needs. This approach promises more effective treatments with fewer side effects, marking a significant advancement in patient care.

The integration of AI in hydrogel development encompasses various stages, each contributing to a holistic framework for material design and optimisation. The AI begins by acquiring comprehensive datasets containing information about hydrogel compositions, fabrication methods, and resulting properties. These datasets serve as the foundation for training AI models. Preprocessing techniques ensure data quality, handling missing values and normalising variables for accurate analyses.

ML algorithms, a subset of AI, play a pivotal role in hydrogel development. Supervised learning techniques, such as regression and classification, enable the prediction of hydrogel properties based on input variables—unsupervised learning, like clustering and dimensionality reduction, aids in identifying patterns and relationships within complex datasets.

AI facilitates the creation of predictive models that map input parameters to specific hydrogel outcomes. These models can forecast properties like swelling behaviour, mechanical strength, and drug release kinetics, allowing researchers to make informed decisions during material design.

AI-driven optimisation methods, including genetic algorithms and Bayesian optimisation, guide the search for optimal hydrogel formulations within vast chemical and structural spaces. These techniques expedite the identification of compositions that meet predefined performance criteria.

While the potential of AI in hydrogel development is vast, several challenges warrant consideration. The availability of high-quality, well-curated datasets is crucial for training robust AI models. Data privacy, the integration of domain knowledge, and the interpretability of AI-generated models are also critical concerns that researchers must address. Additionally, the complexity of hydrogel behaviour, which arises from intricate molecular interactions and crosslinking mechanisms, poses a challenge for accurate predictive modelling.

We would like to point out that this manuscript was prepared with the help of AI-assisted technology. We think that the text’s clarity and coherence were considerably improved via AI-driven language technologies. With the use of these tools, grammatical and structural errors were found and fixed, ensuring that our article meets the strictest requirements for scientific writing. This open disclosure demonstrates our commitment to rigorous and high-level scientific communication. We have given this manuscript the spirit of originality and correctness, which was made possible in part by the cooperation of human expertise and AI-driven language technologies.

This review aims to provide a comprehensive understanding of the potential benefits of AI in designing hydrogels. By shedding light on this innovative approach, we hope to inspire continued research and development, paving the way for more effective targeted therapies that can improve patient outcomes and transform the research of hydrogels in the future.

## 2. Physical and Chemical Methods for Designing Hydrogels

Hydrogels, three-dimensional polymeric networks capable of retaining large amounts of water, have emerged as remarkable materials with a diverse range of applications, particularly in the biomedical and pharmaceutical fields [15]. Their unique properties, such as high water content, biocompatibility, and tuneable mechanical characteristics, make them invaluable for various purposes.

In biomedicine, hydrogels have gained prominence as versatile materials for drug delivery, tissue engineering, wound healing, and diagnostics [15]. These hydrophilic networks can be engineered to mimic the extracellular matrix [16], providing an ideal environment for cell growth and tissue regeneration [17]. Moreover, their ability to encapsulate and release bioactive compounds in a controlled manner has revolutionised drug delivery systems.

In pharmaceutical sciences, hydrogels find applications in drug formulation, where they serve as carriers for poorly water-soluble drugs, enhancing their bioavailability. Additionally, their mucoadhesive properties make them suitable for mucosal drug delivery, opening avenues for novel drug administration routes.

Hydrogels can be prepared using both natural and synthetic materials as precursors. Raw materials, such as cellulose, gelatine, alginate, chitosan (CS), and silk fibroin, are directly sourced from nature and are known for their biocompatibility and bioactive properties. On the other hand, synthetic materials, including polymethylmethacrylate (PMMA), polyurethane (PU), poly(N-isopropylacrylamide) (PNIPAM), poly(lactic acid) (PLA), and poly(lactic-co-glycolic acid) (PLGA), are produced through chemical reactions, offering the advantage of tuneable mechanical properties.

While homopolymeric hydrogels serve a specific purpose, their functionality can be limited. Other biomaterials, such as bioceramics, are often incorporated to enhance the mechanical strength, biodegradability, and/or stimuli-responsiveness of hydrogel matrices. The combination of various biomaterials enables the creation of multifunctional hydrogels that cater to diverse biomedical applications.

This section refers to the physical and chemical methods employed in the design and development of hydrogels. Understanding these methodologies is crucial for harnessing the full potential of hydrogels in addressing contemporary challenges in healthcare and drug delivery. We explore the techniques utilised to tailor hydrogel properties, ensuring they meet the stringent requirements of various biomedical and pharmaceutical applications.

### 2.1. Physical Crosslinking

Physical crosslinking is versatile for creating hydrogels without chemical reactions or covalent bonds. Instead, physical crosslinking relies on non-covalent interactions to form a 3D network, resulting in hydrogels with reversible and dynamic properties. This method offers several advantages, such as ease of preparation, injectability, and the ability to respond to external stimuli, making it suitable for various biomedical and pharmaceutical applications [18].

Some polymers exhibit a temperature-dependent sol-gel transition, forming a gel at a specific temperature range. As the temperature is lowered, these polymers undergo self-assembly, forming a hydrogel network [19]. Common polymers that undergo temperature-induced gelation include thermoresponsive polymers like poly(N-isopropylacrylamide) (PNIPAAm). These hydrogels are particularly attractive for drug delivery applications, as they can respond to changes in body temperature and release drugs accordingly.

Ionic gelation involves using ionic interactions between charged polymer chains and counterions to form a hydrogel network [20]. Examples include alginate and chitosan hydrogels, which can form crosslinks through interactions with divalent cations like calcium ions [20]. The reversibility of these ionic interactions makes them suitable for cell encapsulation and tissue engineering applications.

Certain amphiphilic polymers can self-assemble into hydrogels through hydrophobic interactions or hydrogen bonding [21]. Lipid-based hydrogels, for instance, can spontaneously form through the self-assembly of amphiphilic molecules into nanostructures, resulting in a hydrogel network [22]. These hydrogels have applications in drug delivery, as they can encapsulate hydrophobic drugs and release them in a controlled manner.

Photocrosslinking involves using light to induce the crosslinking of photoreactive molecules or polymers [23]. Photocrosslinkable hydrogels are prepared with photoinitiators that initiate the crosslinking reaction upon exposure to specific wavelengths of light [23]. This method offers precise spatial and temporal control over hydrogel formation, making it valuable for tissue engineering and 3D bioprinting applications.

The freeze–thaw method involves the freezing and thawing of a mixture containing different components, typically polymers and other functional materials, to create a gel-like structure [24]. The process starts by preparing a solvent solution or suspension of the desired components. This mixture is then subjected to a freezing step, where it is cooled to a low temperature, usually below the solvent’s freezing point. During freezing, ice crystals form and the solute molecules, including polymers and other functional materials, are excluded from the ice lattice, increasing their concentration in the unfrozen portion of the solution [25]. After freezing, the sample is thawed, allowing the ice crystals to melt and the components to redistribute in the liquid phase. This process promotes the formation of a gel network as the polymers and other functional materials interact and crosslink, creating a three-dimensional structure that retains a large amount of solvent within its matrix [24,25]. The freeze–thaw cycles can be repeated multiple times to improve the gel’s stability and mechanical properties. By adjusting the composition and freezing–thawing conditions, it is possible to control the hydrophilic/hydrophobic character and other properties of the resulting hybrid hydrogel [25]. This process creates physical crosslinks between the polymer chains, producing a hydrogel.

### 2.2. Chemical Crosslinking

Chemical crosslinking is a versatile and widely used method for obtaining hydrogels with excellent mechanical stability and structural integrity [26]. This process involves the formation of covalent bonds between polymer chains, resulting in a stable 3D network that retains water and forms a hydrogel. Covalent bonds are a type of chemical bond that occurs when two atoms share electrons to achieve a stable electron configuration [27]. In the context of hydrogel formation, when hydrogel precursors, such as polymer chains or monomers, contain functional groups that are capable of forming covalent bonds, a chemical crosslinking process can be initiated [28]. During this process, these functional groups react with one another, establishing covalent bonds between the polymer chains or monomers [29].

Chemical crosslinking is especially suitable for creating hydrogels with controlled porosity, swelling behaviour, and degradation rates, making them ideal for various biomedical and pharmaceutical applications.

In the radical polymerisation method, monomers containing double bonds are polymerised by a crosslinking agent and a radical initiator. The polymerisation reaction generates free radicals, which initiate the chain-growth polymerisation, forming covalent bonds between monomer units [30]. Typical radical initiators include azo compounds and peroxides. The polymerisation process can be carried out in solution or in the presence of a template to create 3D structures [30].

The Michael addition reaction involves the reaction between a Michael donor, typically a thiol group (-SH), and a Michael acceptor, such as an α,β-unsaturated carbonyl compound [31]. This reaction results in the formation of a stable covalent bond, creating crosslinks between polymer chains. Hydrogels formed through Michael’s addition are highly biocompatible and find applications in drug delivery, tissue engineering, and wound healing [32].

Click chemistry refers to a set of high-yield and selective reactions that can efficiently form covalent bonds. Common click chemistry reactions include azide-alkyne cycloaddition, thiol-ene, and tetrazine-norbornene reactions [33]. These reactions are advantageous for hydrogel formation due to their rapid reaction kinetics, high yields, and bioorthogonality, meaning they can be performed in the presence of biological molecules without interfering with cellular processes [34].

Schiff base formation is a chemical reaction between an aldehyde and a primary amine or hydrazine group. This reaction results in the formation of a covalent imine bond, creating crosslinks between polymer chains [35]. Schiff-based hydrogels are often used for drug delivery, as they can release drugs in response to specific environmental cues or triggers [35].

Bioprinting for hybrid hydrogels represents a cutting-edge approach that combines the advantages of both bioprinting and hybrid hydrogel materials [36]. Hybrid hydrogels blend or incorporate multiple materials, such as natural and synthetic polymers [36] or inorganic nanoparticles [37], to create novel hydrogel formulations with enhanced properties. When combined with bioprinting, this approach allows for the precise and controlled deposition of complex 3D structures containing living cells and multifunctional materials [38]. In bioprinting for hybrid hydrogels, the careful selection of bioinks and hybrid materials is crucial. The choice of bioinks is essential to ensure cellular viability, biocompatibility, and mechanical stability [39]. Hybrid materials may include natural polymers like collagen and gelatine, synthetic polyethylene glycol (PEG) and polyvinyl alcohol (PVA), and inorganic nanoparticles like calcium phosphate or gold nanoparticles [40].

Hybrid hydrogels offer a wide range of tuneable mechanical properties. By incorporating different materials with varying stiffness or elasticity, it is possible to create hydrogels that mimic the mechanical properties of native tissues [41]. This is especially important in tissue engineering, where bioprinted constructs must match the target tissue’s mechanical environment for proper cell function and growth [41].

Hybrid hydrogels can be functionalised with bioactive molecules, growth factors, or peptides to promote cell adhesion, proliferation, and differentiation [42]. Bioprinting allows for the precise spatial distribution of these bioactive components within the hydrogel, creating complex microenvironments that can support tissue regeneration and repair.

One of the critical challenges in bioprinting tissue constructs is the lack of vascularisation. Hybrid hydrogels offer a promising solution by incorporating bioactive factors that promote the formation of blood vessels (angiogenesis) [43]. Additionally, hybrid hydrogels can be engineered to contain channels or networks to facilitate the diffusion of nutrients and oxygen, enabling the survival of bioprinted cells within thick tissue constructs [44].

Bioprinting for hybrid hydrogels is a rapidly evolving field with immense potential for advancing tissue engineering, regenerative medicine, and drug development. As researchers continue to innovate in materials science, bioprinting technologies, and tissue engineering, the applications of bioprinted hybrid hydrogels are expected to expand, ultimately leading to groundbreaking advancements in healthcare and personalised medicine. For instance, in regenerative medicine, bioprinted hybrid hydrogels are anticipated to revolutionise the development of patient-specific organoids [45], facilitating drug testing and disease modelling with unparalleled accuracy. Moreover, these hydrogels are set to play a pivotal role in orthopaedics, enabling the creation of custom-designed scaffolds for bone and cartilage repair [46]. In the realm of dermatology, they hold the potential to transform wound healing, with hydrogel-based dressings tailored to individual patient needs [47]. In the pharmaceutical industry, bioprinted hydrogels are envisioned to streamline drug formulation testing, ensuring it becomes safer and more effective [48]. Beyond healthcare, these hydrogels are also expected to find applications in environmental science, such as in the removal of contaminants from water sources [49]. As these innovations gather momentum, they are poised to reshape multiple facets of our lives, promising a future where healthcare and various industries benefit from the limitless potential of bioprinted hybrid hydrogels.

The choice of method depends on the desired properties, functionality, and intended application of the hydrogel. Each technique offers unique advantages and can be tailored to suit specific research or medical needs.

## 3. Numerical and Analytical Methods in Hydrogel Design

Numerical and analytical methods play crucial roles in the design and characterisation of hydrogels. They provide insights into the complex behaviours, properties, and interactions within hydrogel systems.

### 3.1. Numerical Simulations

Numerical simulation methods have emerged as invaluable tools in hydrogel design, revolutionising how researchers approach the development and analysis of these versatile materials. Hydrogels, three-dimensional networks of hydrophilic polymers that can absorb and retain large amounts of water, have found applications in various fields, from biomedical engineering and drug delivery to tissue regeneration. As the demand for hydrogels with tailored properties continues to grow, the integration of computational techniques has become essential for expediting the design process, optimising performance, and predicting behaviour under varying conditions.

The intricate nature of hydrogels, influenced by factors such as polymer composition, crosslinking density, and environmental conditions, presents challenges in accurately characterising and predicting their behaviour solely through traditional experimental approaches. Numerical simulations bridge this gap by providing a virtual laboratory where researchers can explore the intricate interplay between molecular structures, mechanical forces, fluid dynamics, and other critical variables that dictate hydrogel performance. These simulations offer a deeper understanding of hydrogel behaviour, enabling informed design decisions and accelerating the development of hydrogel-based solutions.

Some standard numerical simulation methods used for hydrogel design include Finite Element Analysis (FEA), Computational Fluid Dynamics (CFD), Molecular Dynamics simulations (MD), Monte Carlo Simulations, etc.

FEA is widely used to simulate the mechanical behaviour of hydrogels, including their deformation, stress distribution, and responses to external forces [50,51]. It helps us understand how hydrogels will behave in different loading conditions and assists in designing hydrogels with specific mechanical properties for applications such as tissue engineering, drug delivery, and medical devices.

CFD is used to model the flow of fluids through hydrogel structures [52]. It is important for hydrogels used in drug delivery systems or tissue engineering scaffolds where transporting nutrients, oxygen, and waste products is critical [53]. CFD simulations can provide insights into mass transport phenomena and guide the design of hydrogel structures with optimised fluid flow patterns.

MD simulations are used to study the behaviour of individual molecules within hydrogel networks [54]. They provide insights into the interactions between polymer chains, solvent molecules, and solutes at the atomic level. MD simulations can predict swelling behaviour [55], diffusion rates [56], and biomolecule interactions.

Monte Carlo methods are often employed to model the statistical behaviour of hydrogel systems [57]. These simulations help predict the macroscopic properties of hydrogels based on the behaviour of individual molecules or particles within the system [57]. They can be applied to study phenomena such as the swelling equilibrium, polymer chain conformations, and gel network structure.

Hydrogels are often subjected to multiple physical phenomena simultaneously, such as mechanical deformation, fluid flow, and heat transfer. Multiphysics simulations combine different numerical approaches to model these coupled effects and provide a comprehensive understanding of hydrogel behaviour [58] in complex environments such as tumours [59].

Numerical simulations can be coupled with *optimisation algorithms* to search for the best combination of material properties or structural configurations that meet specific design criteria. This approach is valuable for tailoring hydrogel properties to achieve desired outcomes.

Some simulations combine multiple techniques, such as coupling MD with FEA, to simultaneously capture different aspects of hydrogel behaviour. These hybrid methods allow a comprehensive understanding of complex interactions within hydrogel systems [60].

These numerical simulation methods expedite the design process and enable researchers to explore a vast design space, optimise material properties, and predict hydrogel behaviour across various environments. In combination with experimental data, inverse modelling techniques refine simulations and enhance the accuracy of predictions. Furthermore, the fusion of numerical simulations with optimisation algorithms empowers researchers to identify optimal hydrogel compositions and structures that align with specific performance criteria.

### 3.2. Analytical Methods

The design of hydrogels involves a multifaceted approach that relies heavily on analytical methods to characterise their physical, chemical, and mechanical properties. These methods not only aid in understanding the fundamental behaviour of hydrogels but also drive the optimisation and tailoring of their properties for specific applications. This article delves into the pivotal role of analytical methods in hydrogel design, from characterisation techniques to advanced imaging modalities.

Analysing the chemical composition of hydrogel precursors and networks is crucial for understanding their structure–property relationships. Fourier-transform infrared spectroscopy (FTIR) [61] and nuclear magnetic resonance (NMR) spectroscopy [62] provide insights into functional groups and molecular structures within hydrogel matrices. These methods allow researchers to verify the successful incorporation of desired monomers and crosslinkers and monitor the progress of polymerisation reactions.

Mechanical properties heavily influence the performance of hydrogels in various applications. Compression testing, tensile testing, and rheological analysis quantify parameters like compressive strength, Young’s modulus, and viscosity [63]. These data guide the selection of suitable hydrogel formulations for specific uses, ensuring that mechanical properties align with intended functions.

Hydrogels’ ability to absorb water and swell is a fundamental characteristic that impacts applications such as drug delivery and wound dressings. Swelling behaviour is studied by immersing hydrogels in different solvents and measuring weight changes over time [64]. Analytical balances and swelling ratio calculations provide insights into hydrogel responsiveness to environmental changes, influencing design choices for optimal performance.

Microscopic analysis techniques like scanning electron microscopy (SEM) [65] and atomic force microscopy (AFM) [66] allow researchers to visualise hydrogel surfaces and internal structures. These images reveal information about pore size, distribution, and interconnectedness, which are crucial for applications involving cell adhesion, growth, and the diffusion of therapeutic agents [67].

Thermal properties impact hydrogel stability and behaviour at different temperatures. Differential scanning calorimetry (DSC) [68] and thermogravimetric analysis (TGA) [69] enable the assessment of glass transition temperatures, melting points, and thermal degradation profiles. Such data aid in determining suitable processing conditions and the temperature ranges within which hydrogels maintain their integrity.

Analytical methods are pivotal in studying drug release kinetics from hydrogel matrices. UV-Vis spectroscopy [70] and high-performance liquid chromatography (HPLC) monitor the concentration of released substances over time. These data are crucial for designing hydrogel-based drug delivery systems with controlled and sustained release profiles [71].

Recent advancements have introduced sophisticated imaging methods such as confocal microscopy and magnetic resonance imaging (MRI) to further probe hydrogel behaviour [72]. These techniques allow the in-depth visualisation of hydrogel interactions with cells, tissues, and drugs, providing insights into real-time responses and interactions.

#### Statistical Data Analysis

**Figure 1:**
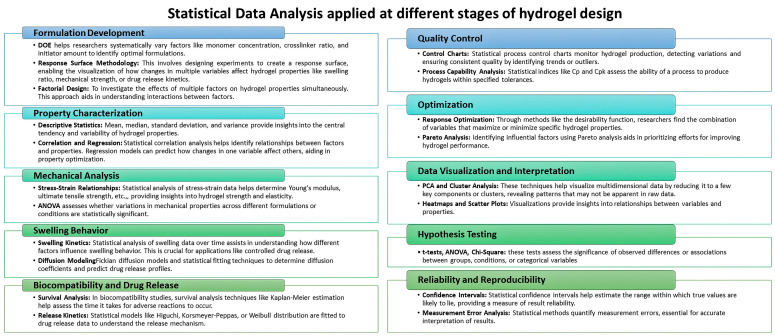
Statistical data analysis at different stages of hydrogel design.

Statistical data analysis involves applying various statistical techniques to process, interpret, and draw meaningful conclusions from experimental data. In hydrogel design, statistical analysis plays a crucial role in understanding the relationships between different variables, optimising formulations, and ensuring the reproducibility of results. Statistical data analysis methods are applied at various stages of hydrogel design; see Figure 1.

Before conducting experiments, researchers use statistical tools to design experiments effectively. Techniques like Design of Experiments (DOE) help to determine which variables to control, which to manipulate, and how many experiments to perform to gather sufficient data.

Statistical analysis starts with the collection of data from experiments. These data can include information about hydrogel composition, structure, mechanical properties, swelling behaviour, drug release profiles, and more.

Descriptive statistics provide a summary of the collected data. Measures like mean, median, standard deviation, and range give an overview of the central tendency and variability of the data.

Researchers use correlation analysis to identify relationships between different variables. For example, it can reveal if there is a correlation between the composition of the hydrogel and its mechanical strength [73].

Regression models help establish mathematical relationships between variables. Researchers can use linear or nonlinear regression to predict one variable based on the values of others. This is useful for predicting hydrogel behaviour under different conditions [74].

Analysis of Variance (ANOVA) assesses the variance between different groups or conditions. It helps determine if the variations observed in the data are significant and whether they are due to manipulated variables or random chance [75].

Principal Component Analysis (PCA) is a dimensionality reduction technique that transforms complex data into a lower-dimensional space. It can help identify patterns and trends in multi-dimensional data sets, making it useful for analysing complex hydrogel datasets [76].

Multivariate analysis involves the analysis of multiple variables simultaneously to uncover hidden patterns and relationships that might not be apparent in individual studies [77].

Statistical methods are employed to ensure the quality and consistency of hydrogel production. Control charts, process capability analysis, and six sigma methodologies help maintain the desired quality standards.

Statistical analysis assesses the reliability and reproducibility of hydrogel properties allowing the calculation of confidence intervals, the assessment of experimental errors, and the determination of the precision of measurements.

Researchers use statistical optimisation techniques to find the optimal combination of hydrogel parameters that yield the desired properties. This is particularly useful in fine-tuning hydrogel formulations for specific applications.

Visualising data through plots, graphs, and charts helps us to understand trends and patterns intuitively. Visualisation tools (scatter plots, line charts, bar charts, heatmaps, 3D surface plots, radial charts, box plots, principal component analysis plots) enhance the communication of results and aid in decision-making.

Statistical tests, such as t-tests [78] or chi-square tests [79], are used to test hypotheses and determine whether observed differences between groups are statistically significant.

In hydrogel design, statistical data analysis aids in making informed decisions, optimising formulations, understanding the effects of variables, and ensuring the reliability of results. By applying appropriate statistical techniques, researchers can uncover insights that guide the development of hydrogels with tailored properties for a wide range of applications.

## 4. Leveraging Artificial Intelligence in Hydrogel Design

Leveraging AI in hydrogel design involves utilising advanced computational techniques to optimise and accelerate the development of hydrogel materials with specific properties and functionalities. AI-driven approaches revolutionise conventional trial-and-error methods, enabling rapid and informed material design. This leads to the development of hydrogels better tailored for diverse applications in medicine and biology [80]. Below, we present some expanded details and explore how AI is applied to the design and selection of hydrogels.

In the case of material design and selection, AI algorithms can predict and optimise hydrogel properties based on desired characteristics. ML models analyse large datasets of material properties to guide the selection of polymers, crosslinkers, and additives for specific applications. In addition, ML models can predict hydrogel behaviours, such as swelling ratios, mechanical strength, and degradation rates, based on formulation and environmental conditions.

AI can perform virtual screenings of vast chemical spaces to identify potential monomers, crosslinkers, and reaction conditions for synthesising hydrogels with desired properties, reducing the need for extensive experimental trial and error.

Also, AI-driven optimisation algorithms can enhance the hydrogel synthesis process by adjusting parameters such as temperature, pH, reaction time, and swelling to maximise yield and desired properties. For example, Islamkulov et al. used AI-supported optimisation applications (multilayer neural network sigmoid function model) for determining the swelling kinetics of hydrogel networks. In addition, the results of swelling behaviour under different experimental conditions, such as different crosslinker concentration temperatures and salt solutions, provided a deeper understanding of the physicochemical properties of the prepared hydrogels [81]. An important parameter for achieving a reproductible hydrogel is the gelation kinetics. AI models can predict hydrogel gelation kinetics by analysing the kinetics of polymerisation reactions, aiding in controlling gelation time and achieving reproducible results [82].

AI-enabled image analysis and spectroscopic techniques assist in the characterisation of hydrogel structures, porosity, and mechanical properties, ensuring quality and consistency in production. These analyses can be performed by using advanced imaging techniques such as scanning microscopy or NMR spectroscopy.

AI algorithms can assess the biocompatibility and functionality of hydrogels for specific biological applications, guiding the design of hydrogels for drug delivery, tissue engineering, and wound healing. Boztepe and colleagues [12] introduced an innovative hydrogel for the controlled release of doxorubicin. This research addressed a notable gap in the exploration of AI-driven hydrogel systems. Their study revealed the remarkable performance of the AI-based model in accurately predicting the drug release behaviour of the hydrogels they developed. These findings underscore the significance of such investigations in advancing novel materials while building upon empirical knowledge.

AI-driven data analysis can reveal previously unnoticed patterns (correlations between components, optimal manufacturing conditions, material interactions, performance over time, cost-effective formulations, biological response) and relationships within hydrogel datasets, leading to the discovery of novel hydrogel formulations and applications. These unnoticed patterns are often buried within vast datasets and can be challenging for humans to discern. AI’s strength lies in its ability to sift through immense amounts of data, and to make predictions or recommendations based on these findings.

Another essential aspect of the research and development of hydrogels is the number of experimental iterations required. AI can accelerate hydrogel development, leading to the faster translation of hydrogel-based technologies, from research to practical applications.

AI-driven materials informatics platforms organise and analyse hydrogel-related data, facilitating collaboration and knowledge-sharing among researchers. Also, AI can help design hydrogels with tailored properties for specific patient needs, such as wound dressings or drug delivery systems.

AI-driven predictive models assist in predicting hydrogel performance under different regulatory conditions, aiding in compliance with safety and efficacy standards.

Integrating AIs into hydrogel research and development streamlines processes; it accelerates innovation and enhances the capabilities of hydrogels for diverse biomedical applications.

## 5. Machine Learning Techniques in Hydrogel Development

ML techniques have sparked a paradigm shift in the realm of hydrogel development. These sophisticated computational tools are reshaping how researchers approach materials design and expediting the entire innovation lifecycle of hydrogels. By harnessing the power of data-driven insights and predictive modelling, ML techniques have established themselves as indispensable assets at various crucial stages of hydrogel development, paving the way for accelerated discovery, enhanced precision, and the creation of novel and tailored hydrogel materials.

### 5.1. Machine Learning Subsets

ML subsets can be applied to various hydrogel research and development aspects, offering innovative solutions and accelerating progress in this field. ML consists of several subsets or branches [83], each with its own focus and techniques. Some of these subsets include:

Supervised Learning: In hydrogel research, supervised learning involves training a model on labelled data, where inputs (e.g., polymer type, crosslinking density) are associated with desired outputs (e.g., swelling ratio, degradation rate). This approach enables the prediction of hydrogel properties based on known relationships, aiding in efficiently screening potential formulations and optimising synthesis conditions [84].

Unsupervised Learning: Unsupervised learning techniques like clustering can uncover hidden patterns within complex hydrogel datasets. By grouping similar hydrogels based on structural and functional attributes, researchers can identify novel categories or classes of hydrogels with distinct behaviours, facilitating targeted investigations and customised designs [85].

Semi-Supervised Learning: When hydrogel datasets have limited labelled samples, semi-supervised learning combines labelled and unlabelled data. This approach can enhance predictions by leveraging the broader dataset, providing valuable insights into hydrogel behaviour even with a scarcity of labelled samples.

Reinforcement Learning: Hydrogel design can benefit from reinforcement learning by treating it as a sequential decision-making process. Algorithms can optimise synthesis parameters over multiple iterations to achieve the desired properties, such as mechanical strength or drug release profiles, while considering the feedback received from previous experiments [86].

Deep Learning: Deep neural networks, a subset of AI [87], can capture intricate relationships between input variables and hydrogel properties. By training on a diverse range of hydrogel compositions and experimental outcomes, deep learning models can predict complex behaviours, guiding the design of new hydrogel formulations.

Transfer Learning: Transfer learning allows models pre-trained on one hydrogel dataset to be fine-tuned for a different application. For instance, a neural network initially trained to predict swelling behaviour in one type of hydrogel can be adapted to predict degradation in another, saving time and computational resources [88].

Generative Adversarial Networks (GANs): GANs can aid in the design of new hydrogel structures by generating molecular configurations that meet specific performance criteria. This approach is promising for creating unique hydrogel formulations optimised for biomedical applications [89].

**Figure 2:**
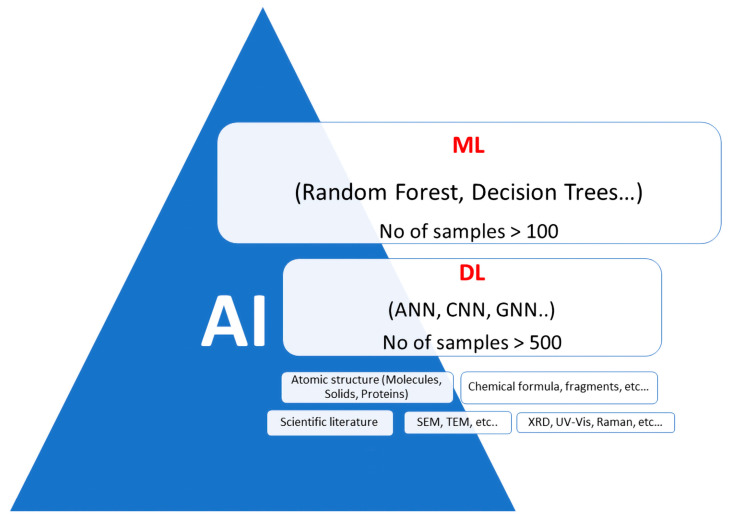
Schematic showing an overview of AI, ML, and DL methods.

Applying these ML subsets to hydrogel research offers a multidimensional approach to understanding, designing, and optimising hydrogel materials for diverse biomedical, pharmaceutical, and industrial purposes. A schematic of AI-ML context and some of the application areas in the field of hydrogels are shown in Figure 2.

### 5.2. Machine Learning Algorithms

#### 5.2.1. Random Forest

RF stands out as a powerful and versatile ML algorithm that has revolutionised the field of hydrogel development [10]. Built upon the principles of ensemble learning, RF offers a sophisticated solution for tackling complex challenges in materials design, property prediction, and optimisation. Its unique characteristics make it an invaluable asset in pursuing innovative and tailored hydrogel materials.

**Figure 3:**
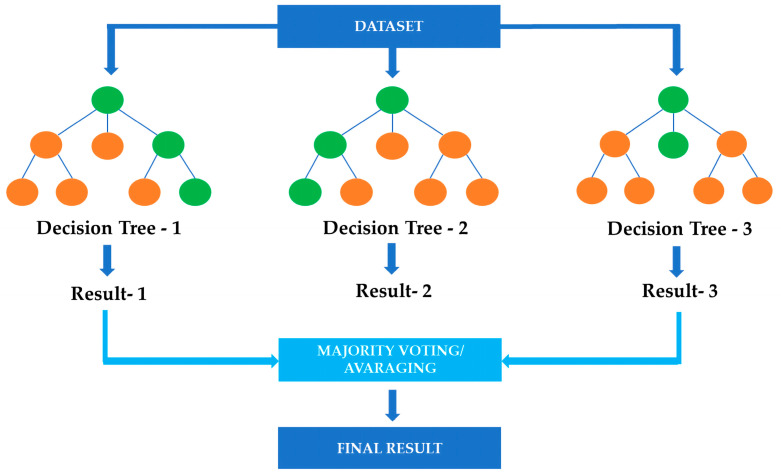
Simplified random forest.

At its core, RF is a collection of decision trees that operate collectively as a cohesive unit. Each decision tree is constructed using a random subset of the available data and features, making them diverse and distinct [90]. This diversity is a crucial strength of RF, enabling the model to capture a wide range of relationships within the data, from simple to complex. When a prediction is required, each decision tree contributes its output, and the final result is determined by aggregating these outputs—usually through voting for classification tasks or averaging for regression tasks (Figure 3). This ensemble approach enhances RF’s accuracy, stability, and robustness, enabling it to handle the noisy or incomplete datasets that often characterise hydrogel research.

RF’s ability to handle high-dimensional data and complex interactions is advantageous in hydrogel development. As researchers work with intricate combinations of variables—from monomer types and crosslinking ratios to environmental conditions and desired material properties—RF excels at identifying non-linear relationships and interactions that could be challenging to discern through traditional methods. By analysing these relationships comprehensively, RF aids researchers in predicting how changes in one or more variables may impact the final hydrogel properties, thus guiding more informed and efficient decision-making.

Moreover, RF offers a degree of interpretability that differentiates it from other ML techniques. It can provide insights into feature importance, revealing which variables contribute most significantly to the model’s predictions. This feature importance analysis in hydrogel research is invaluable for uncovering the key factors influencing specific material properties or behaviours. By identifying the most influential parameters, researchers can focus on fine-tuning these aspects of hydrogel design to achieve the desired outcomes effectively.

The RF method was recently applied for the hypothesis that polysaccharide hydrogels may feature fundamental separation criteria relevant to the permeability of compounds across the Gram-negative bacterial cell envelope, and that such permeability data could be used for predicting antibiotic accumulation in such bacteria [91]. Applying contemporary ML tools to the in vitro data, the same authors reported the first data on in bacterio accumulation of aminoglycosides and sulphonamides—essential classes of antibiotics used to treat Gram-negative infections. Expanding the investigations to antibiotic activity against highly relevant Gram-negative species gave evidence that in vitro permeability data may allow the exclusion of inactive substances at an early stage of antibiotic development [91].

#### 5.2.2. Artificial Neural Network

Artificial Neural Networks (ANNs), inspired by the human brain’s neural structure, excel at capturing intricate patterns in large datasets [83]. In hydrogel research, ANNs can model complex hydrogel–property relationships, allowing for accurate predictions of material behaviours. ANNs have been employed to optimise hydrogel formulations, predict drug release kinetics, and even simulate hydrogel–cell interactions, accelerating the understanding of hydrogel functionality.

For example, Brahima et al. used an ANN to model the nonlinear, multivariable, and complex drug delivery behaviour of poly(NIPAAm–co–AAc) IPN hydrogel systems. The developed ANN model was used to efficiently predict the drug release behaviours of hydrogels [92].

#### 5.2.3. Support Vector Machines

Support Vector Machines (SVM) are a supervised learning method used for classification and regression tasks [93]. In hydrogel development, SVM can classify hydrogel formulations based on their properties or predict properties based on known compositions. SVM aid in identifying relevant features that influence hydrogel performance, guiding researchers to prioritise specific components for achieving desired outcomes.

#### 5.2.4. Deep Neural Networks

Deep Neural Networks (DNNs) can predict hydrogel properties based on their molecular structures [83,94]. By training on a dataset of known hydrogel compositions and their corresponding properties, DNNs can learn complex relationships between molecular features and hydrogel behaviour. This can aid in predicting properties like mechanical strength, swelling behaviour, and drug release profiles for new hydrogel formulations. DNNs can also be used for optimising hydrogel synthesis parameters. By setting up the DNN as an optimisation algorithm, it can iteratively suggest modifications to the formulation based on desired property outcomes. This can accelerate the process of finding optimal synthesis conditions.

#### 5.2.5. Convolutional Neural Networks

Convolutional Neural Networks (CNNs) excel at analysing visual data, making them helpful in analysing hydrogel images, such as microstructure images captured through microscopy. CNNs can identify features, patterns, and structures in these images, providing insights into the internal structure of hydrogels [94].

In the context of 3D-printed hydrogels, CNNs can analyse the printing process and optimise printing parameters. By learning from 3D printing data, CNNs help the user adjust the printing speed, material deposition rate, and other variables to achieve the desired printing outcomes [95]. For example, a deep learning model using CNN was used to generate a model that differentiated between excellent and poor hydrogel prints. The CNN model was found to classify the bad and good images with an accuracy of 93.51%. The model achieved a validation accuracy of 90.244% [96]. Jin et al. [68] developed an anomaly detection system to classify imperfections for hydrogel-based bioink based on convolutional neural networks. Images were processed as small image patches for grid, gyroid, rectilinear, and honeycomb shapes. This research envisions high-quality tissue composition through real-time autonomous correction in the 3D bioprinting process [97].

CNNs can assist in designing microfluidic channels for hydrogel synthesis. By considering fluid flow dynamics, mixing efficiency, and gelation behaviour, CNNs can suggest optimised channel geometries for efficient and controlled synthesis.

CNNs can be employed to analyse and characterise the surface features of hydrogels. This includes identifying surface roughness, pore size distribution, and other topographical aspects influencing hydrogel performance.

Also, this algorithm can aid in quality control by identifying defects or inconsistencies in hydrogel products. This can ensure that the synthesised hydrogels meet the desired specifications and perform as expected.

The versatility of the above-mentioned algorithms can be extended. Combining some of the above-mentioned can aid researchers in identifying the most relevant variables among a large pool of options. This streamlines hydrogel development by focusing on the most impactful factors, reducing experimentation time and resources. For example, Pluronic F127, Pluronic F68, and Methocel K4M created and characterised enemas that deliver rectal protein. The concentrations of various polymers were utilised as input values to correlate with the final properties of the hydrogel using FormRules version 4.03, a commercial hybrid artificial intelligence tool platform that combines ANNs and fuzzy logic technologies. It is possible to assess the effects of each polymeric component in the hydrogel composition. For instance, it was discovered that F127 affected mucoadhesion and syringeability [98].

In a recent study conducted by Boztepe et al., ANNs were used to predict doxorubicin delivery from pH- and temperature-responsive poly(N–Isopropyl acrylamide-co-Acrylic acid)/Poly(ethylene glycol) (poly(NIPAAm-co-AAc)/PEG) interpenetrating polymer network (IPN) hydrogel [12]. In the same study conducted by Boztepe et al., derivates of SVM were used to predict doxorubicin delivery from pH- and temperature-responsive IPN hydrogel [12].

Another study employed random forests, Gaussian process, and support vector machines as ML models to predict the cardiomyocyte (CM) content following the differentiation of human-induced pluripotent stem cells (hiPSCs) encapsulated in hydrogel microspheroids and to identify the main experimental variables affecting the CM yield. The models were built to predict two classes, sufficient and insufficient, for CM content on differentiation day 10. The best model predicted the sufficient class with an accuracy of 75% and a precision of 71%. This study showed that we can extract information from the experiments and build predictive models that could enhance the cell production by using ML techniques [99].

In a novel study, Li. et al. showed a combinational approach to generate a structurally diverse hydrogel library with more than 2000 peptides and evaluated their corresponding properties. The authors combined algorithms with the best precisions (54%, 57%, and 62% for RF, logistic regression, and gradient boosting, respectively, at the 50% recall). The authors correlated chemical variables and quantitative structure–property interactions with the self-assembly behaviour, and they were effective in identifying key structural elements influencing hydrogel formation [100].

Moreover, many of these algorithms can be combined for hydrogel synthesis. In a recent work, a CNN regression method was used to predict the Young’s modulus and Poisson’s ratio of BG-COL composites. First, 2000 images of BG-COL microstructures were generated. Then, the mechanical properties of the BG-COL composite were calculated using the finite element simulation. This numerical simulation software obtained data that were used to train a CNN regression model for predicting the mechanical properties of BG-COL based on its microstructural image. The authors demonstrated that the accepted CNN regression model could predict the mechanical properties of BG-COL. Hence, it can aid in overcoming the challenges of predicting these properties with traditional homogenisation methods. This work could guide the design of BG-COL and other composite hydrogels [50].

Zhu et al. used DNN and 3D CNN to reveal the implicit relationship between the network structure and mechanical properties of hydrogels to predict mechanical properties from different network structures. A modelling method for a single-network hydrogel network, that is, a self-avoiding walk network model which approximates the real polyacrylamide (PAAm) hydrogel structure at a mesoscopic scale, was proposed. After, the authors developed a DNN based on MLP and a 3D CNN containing the physical information of the network and utilised them to predict the nominal stress–stretch curves of hydrogels under uniaxial tension. By having a dataset of 2200 randomly generated network structures of PAAm hydrogel and their corresponding stress–stretch curves, the authors trained and evaluated the performance of the two models [94].

The selection of machine learning algorithms in hydrogel development is a dynamic and data-driven process. Researchers must consider the specific objectives, data types, and desired outcomes when choosing the most appropriate and particular machine learning algorithm or technique. This adaptability and versatility make AI a powerful tool in advancing the development of hydrogels for a wide range of biomedical applications.

## 6. Conclusions and Future Perspectives

Hydrogel-based strategies hold great promise and offer customisable solutions for various clinical scenarios. Hydrogels have immense promise for transforming hard and soft tissue treatments, but their successful implementation requires overcoming various obstacles through continued research, collaboration, and regulatory compliance.

Numerical simulations offer a virtual playground where hydrogel properties can be tailored, refined, and fine-tuned, creating a synergy between computational and experimental methodologies. As the field of hydrogel design continues to evolve and advance, the integration of numerical simulations can expand the frontiers of what is achievable with these remarkable materials.

Looking ahead, the fusion of AI with hydrogel development holds immense promise. Advanced AI techniques, including deep learning and reinforcement learning, will likely push the boundaries of predictive accuracy and material optimisation. Collaborations between materials scientists, chemists, and AI experts will foster interdisciplinary innovation, leading to the discovery of novel hydrogel formulations with tailored functionalities.

Across the hydrogel landscape, ML’s transformative influence is undeniable. As researchers strive to design hydrogels with specific properties and functionalities, traditional methods often entail time-consuming trial-and-error approaches. In contrast, ML techniques are potent engines for pattern recognition, data analysis, and predictive modelling. They enable researchers to navigate the complex interplay of numerous variables—such as hydrogel composition, processing parameters, and end-use requirements—by generating comprehensive insights from large, intricate datasets. This capability propels hydrogel development beyond the boundaries of conventional experimentation, allowing researchers to extract valuable knowledge from raw data and make informed decisions with unprecedented efficiency. The continued collaboration between materials scientists, chemists, and AI/ML experts is instrumental in advancing hydrogel design. Together, they can harness the power of data-driven approaches, tackle complex problems, and create innovative hydrogel-based solutions that have a profound impact on healthcare and biomedical applications.

ML’s impact is particularly profound in optimising hydrogel formulations. By rapidly evaluating an extensive range of chemical compositions and structural arrangements, ML algorithms guide researchers towards promising hydrogel candidates for specific applications. This ability to traverse multidimensional parameter spaces accelerates the identification of optimal formulations, expediting the path from conceptualisation to tangible hydrogel prototypes. Furthermore, ML techniques empower researchers to predict hydrogel properties with remarkable accuracy, sparing them the need for resource-intensive trial iterations. This predictive prowess shortens development timelines and empowers researchers to fine-tune hydrogel properties to precise specifications—a critical advantage in tailoring materials for diverse applications, from drug delivery systems to tissue engineering scaffolds.

Integrating ML techniques into hydrogel development represents a fundamental shift from conventional approaches to a dynamic, data-centric methodology. ML’s capacity to handle complexity, recognise intricate patterns, and optimise outcomes positions it as an indispensable tool in the arsenal of modern hydrogel development. As the boundaries of ML continue to expand, its synergy with hydrogel research is poised to reshape the trajectory of biomaterials innovation.

As ML continues to evolve, advanced techniques like deep learning and reinforcement learning are promising for further pushing push the boundaries of hydrogel research. Integrating domain knowledge into ML models and addressing challenges such as data scarcity and interpretability will be crucial for realising the full potential of ML in hydrogel development.

In conclusion, integrating ML techniques such as RF, ANN, SVM, and LR has revolutionised the field of hydrogel development. These tools expedite the discovery process, optimise material properties, and pave the way for innovative applications in drug delivery, tissue engineering, and diagnostics. As ML technology advances, it is poised to reshape the landscape of hydrogel research, unlocking new possibilities and accelerating advancements in biomaterials science.

Even though incorporating AI into hydrogel design has opened a new era of precision and efficiency, significant challenges persist, necessitating innovative solutions and interdisciplinary collaboration. As we mentioned, AI models rely heavily on data. In hydrogel design, comprehensive and accurate datasets can be elusive. Materials scientists, chemists, and AI experts must collaborate to curate high-quality data [101,102].

Successful hydrogel design demands input from chemistry, biology, materials science, and AI. Effective cross-disciplinary collaboration is essential but can be challenging. Hydrogel properties are influenced by numerous factors. Modelling these interactions accurately remains a challenge [101,102].

As AI-driven hydrogel design advances, ethical concerns surrounding data privacy, bias in algorithms, and intellectual property rights become more prominent. Addressing these issues transparently and ethically is critical to maintaining trust and integrity in research. Transitioning from small-scale AI-optimised designs to large-scale production is challenging, particularly for medical and industrial applications [101,102].

Hydrogels developed using AI may face regulatory hurdles, particularly in the medical field. However, demonstrating their safety and efficacy to regulatory bodies is a complex and resource-intensive process [101,102].

As AI continues to evolve, its integration with hydrogel research holds the promise of unlocking new capabilities and applications, revolutionising the field of biomaterials and shaping the future of medical science and technology.
